# EEG microstate quantifiers and state space descriptors during anaesthesia in patients with postoperative delirium: a descriptive analysis

**DOI:** 10.1093/braincomms/fcad270

**Published:** 2023-10-17

**Authors:** Bruno Neuner, Simone Wolter, William J McCarthy, Claudia Spies, Colm Cunningham, Finn M Radtke, Martin Franck, Thomas Koenig

**Affiliations:** Department of Anaesthesiology and Intensive Care Medicine, Charité-Universitätsmedizin Berlin, Corporate Member of Freie Universität Berlin and Humboldt Universität zu Berlin, 10117 Berlin, Germany; Department of Anaesthesiology and Intensive Care Medicine, Charité-Universitätsmedizin Berlin, Corporate Member of Freie Universität Berlin and Humboldt Universität zu Berlin, 10117 Berlin, Germany; Centre for Cancer Prevention and Control Research, Fielding School of Public Health and Jonsson Comprehensive Cancer Centre, University of California Los Angeles (UCLA), Los Angeles, CA 90095-1781, USA; Department of Anaesthesiology and Intensive Care Medicine, Charité-Universitätsmedizin Berlin, Corporate Member of Freie Universität Berlin and Humboldt Universität zu Berlin, 10117 Berlin, Germany; School of Biochemistry and Immunology, Trinity Biomedical Sciences Institute & Trinity College Institute of Neuroscience, Trinity College Dublin, 2 D02 R590 Dublin, Ireland; Department of Anaesthesiology and Intensive Care Medicine, Charité-Universitätsmedizin Berlin, Corporate Member of Freie Universität Berlin and Humboldt Universität zu Berlin, 10117 Berlin, Germany; Department of Anaesthesia and Intensive Care, Hospital of NykÃ¸bing Falster, Fjordvej 15, 4800 NykÃ¸bing Falster, Denmark; University of Southern Denmark (SDU), Campusvej 55, 5230 Odense, Denmark; Department of Anaesthesiology and Intensive Care Medicine, Charité-Universitätsmedizin Berlin, Corporate Member of Freie Universität Berlin and Humboldt Universität zu Berlin, 10117 Berlin, Germany; Department of Anaesthesia, Alexianer St.Hedwig Hospital, 10115 Berlin, Germany; University Hospital of Psychiatry, Translational Research Centre, University of Bern, 3000 Bern, Switzerland

**Keywords:** multichannel EEG, anaesthesia, postoperative delirium, EEG microstates, EEG state space descriptors

## Abstract

Postoperative delirium is a serious sequela of surgery and surgery-related anaesthesia. One recommended method to prevent postoperative delirium is using bi-frontal EEG recording. The single, processed index of depth of anaesthesia allows the anaesthetist to avoid episodes of suppression EEG and excessively deep anaesthesia. The study data presented here were based on multichannel (19 channels) EEG recordings during anaesthesia. This enabled the analysis of various parameters of global electrical brain activity. These parameters were used to compare microstate topographies under anaesthesia with those in healthy volunteers and to analyse changes in microstate quantifiers and EEG global state space descriptors with increasing exposure to anaesthesia. Seventy-three patients from the Surgery Depth of Anaesthesia and Cognitive Outcome study (SRCTN 36437985) received intraoperative multichannel EEG recordings. Altogether, 720 min of artefact-free EEG data, including 210 min (29.2%) of suppression EEG, were analysed. EEG microstate topographies, microstate quantifiers (duration, frequency of occurrence and global field power) and the state space descriptors sigma (overall EEG power), phi (generalized frequency) and omega (number of uncorrelated brain processes) were evaluated as a function of duration of exposure to anaesthesia, suppression EEG and subsequent development of postoperative delirium. The major analyses involved covariate-adjusted linear mixed-effects models. The older (71 ± 7 years), predominantly male (60%) patients received a median exposure of 210 (range: 75–675) min of anaesthesia. During seven postoperative days, 21 patients (29%) developed postoperative delirium. Microstate topographies under anaesthesia resembled topographies from healthy and much younger awake persons. With increasing duration of exposure to anaesthesia, single microstate quantifiers progressed differently in suppression or non-suppression EEG and in patients with or without subsequent postoperative delirium. The most pronounced changes occurred during enduring suppression EEG in patients with subsequent postoperative delirium: duration and frequency of occurrence of microstates C and D progressed in opposite directions, and the state space descriptors showed a pattern of declining uncorrelated brain processes (omega) combined with increasing EEG variance (sigma). With increasing exposure to general anaesthesia, multiple changes in the dynamics of microstates and global EEG parameters occurred. These changes varied partly between suppression and non-suppression EEG and between patients with or without subsequent postoperative delirium. Ongoing suppression EEG in patients with subsequent postoperative delirium was associated with reduced network complexity in combination with increased overall EEG power. Additionally, marked changes in quantifiers in microstate C and in microstate D occurred. These putatively adverse intraoperative trajectories in global electrical brain activity may be seen as preceding and ultimately predicting postoperative delirium.

## Teaser

Neuner *et al*. analysed intraoperative multichannel EEGs. With increasing exposure to anaesthesia, EEG microstate quantifiers and state space descriptors (parameters of global brain activity) showed different trajectories in non-suppression and suppression EEG and in patients with or without subsequent postoperative delirium. Their role in predicting postoperative delirium warrants further research.

## Graphical Abstract

**Graphical Abstract:**
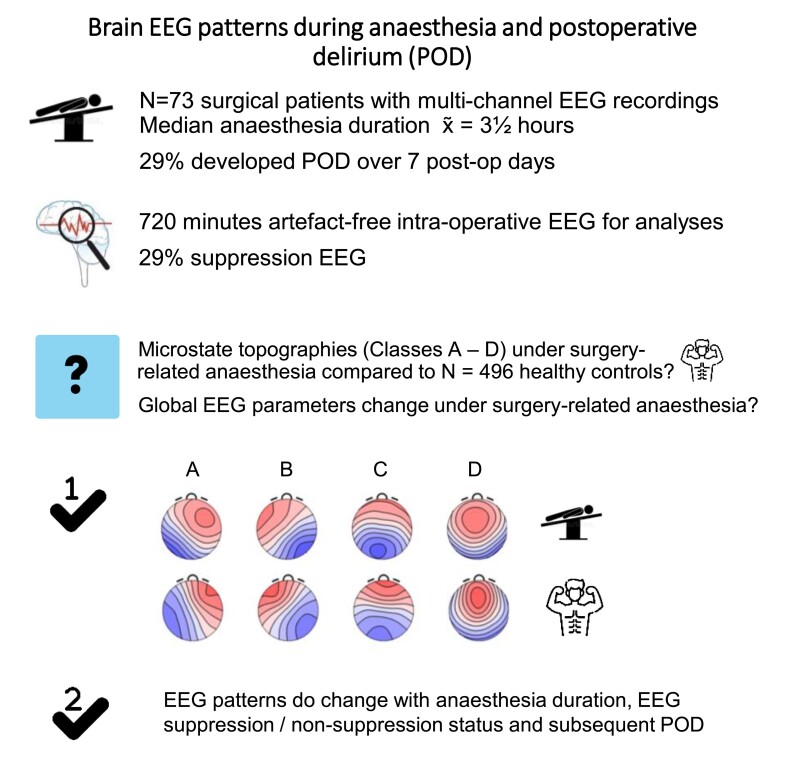

## Introduction

Anaesthesia is defined as a reversible loss of consciousness, sensation and awareness, induced for medical purposes. The maintenance of general anaesthesia is routinely monitored using peripheral parameters such as oxygenation, ventilation, circulation and temperature.^1^ In recent years and especially in older patients, it became common to additionally monitor electrical activity of the brain through processed EEG measures.^2^ Often a bi-frontal two- or four-channel EEG recording is used, generating, apart from the raw EEG trace, a single measure of depth of anaesthesia (‘processed EEG’ index).^2,3^ One reason for this development is the growing number of surgeries in older patients^4,5^ who are at increased risk of developing postoperative delirium (POD).^6,7^ POD typically occurs from minutes to several days after the surgery. More specifically, this is an acute state with disturbances in attention, awareness and cognition, representing a decline from the patient’s preoperative mental status.^8^ Processed EEG monitoring techniques allow the anaesthesiologist to keep the depth of anaesthesia within a predefined range, to avoid excessively deep anaesthesia and burst suppression EEG—an EEG pattern characterized by periods of high-voltage electrical activity alternating with periods of very low electrical activity^9^—and thus to decrease the risk of POD.^10-12^ A number of further EEG measures and indices, based on bi-frontal intra- and perioperative EEG recordings, were evaluated in regard to POD prediction.^13-15^ But even if the bi-frontal EEG recording is able to quantify depth of anaesthesia and to detect intervals of suppression EEG, this approach fails to capture the whole-brain electrical activity. For the wakeful active state, parallel processing in large brain neural networks and even inherent global brain activity at rest seem to be the norm.^16^ This whole-brain pattern of electrical activity—detectable through multichannel EEG recordings—not only co-varies with the functional state of the healthy brain^17^ and with different states of consciousness^18,19^ but likewise with disease conditions affecting cognitive and other higher brain functions.^20^ It is probably a defective whole-brain pattern of electrical activity and defective global brain network activity that contributes to delirium.^21^

**Figure 1:**
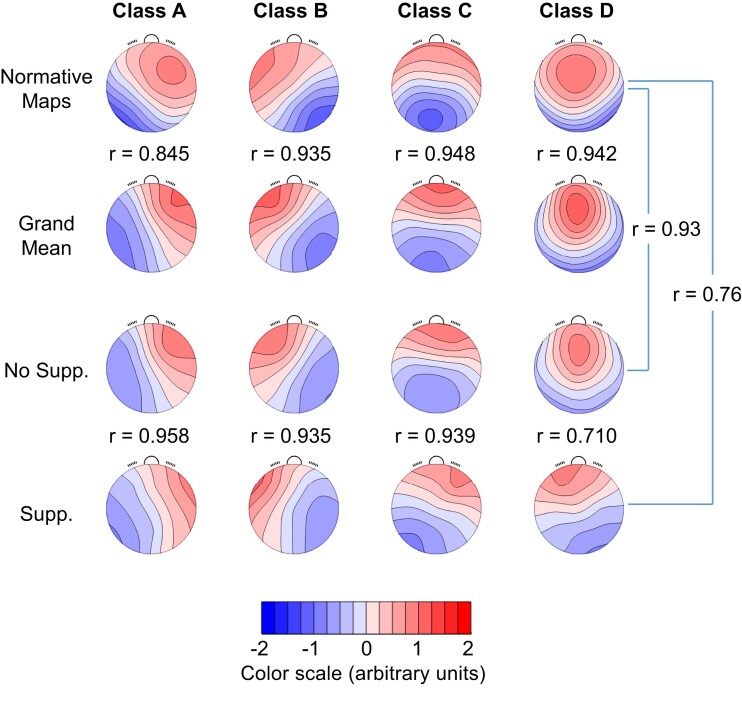
**Microstate topographies in *n* = 496 healthy volunteers (normative data) and in *n* = 73 patients exposed to anaesthesia during surgery.** Row 1 depicts the four-microstate solution in normative data.^22^ The four-microstate solution after exposure to anaesthesia is shown in Rows 2–4. Row 2 depicts the grand means of all 885 EEG intervals. The grand means in non-suppression intervals are depicted in Row 3 and those in suppression intervals in Row 4. Numbers indicate correlations between all four microstate classes (*r*-values within the figure) and between normative microstate D and microstate D after exposure to anaesthesia (*r*-values on the right side of the figure). Microstate D has been associated with the attention-related brain network in previous studies. Supp., suppression.

One method of analysing whole-brain electrical activity is the analysis of microstates, i.e. non-overlapping sub-second EEG periods of quasi-stable field topography.^22-25^ These transient and global states of common-phase oscillations are measured on the 3D scalp^16,23^ and represent the dynamic assembling of large-scale intracranial neural networks.^16,25^ Their scalp potential distributions can be visualized as ‘microstate maps’ or ‘microstate topographies’.^16^ Microstates assumingly represent global states of neuronal co-activations ^17,22,26^ and appear to be rather common across individuals and function states.^27,28^ Microstates can be efficiently grouped into a small set of classes defined by common topographies. While the optimal number of topographies needs to be determined for any specific study population, e.g. by analysing the explained variance for solutions with different numbers of microstate classes, a four-microstate solution is often used.^16^ In awake subjects and using combined fMRI and EEG analyses, these four ‘canonical’ microstates (see the first row in Fig. 1) were empirically attributed to specific resting state networks. Although such ‘one-to-one attributions of microstates to brain functions based on fMRI correlations must be made with caution’,^16^ the four different microstates were linked to the auditory network (microstate A), to the visual network (microstate B), to the default mode or task-negative network (microstate C) and to the attention network (microstate D).^16,29^ In addition to the spatial pattern of the microstates, their temporal patterns may be analysed with a variety of quantifiers, such as microstate ‘duration’ (how long a microstate remains stable, usually between 80 and 120 ms^22^), ‘frequency of occurrence’ within a specific interval in time and ‘global field power’ (GFP, a measure of the magnitude of the synchronous activation of large numbers of intracranial neurons^30^).

Another method of analysing whole-brain electrical activity is the analysis of state space descriptors.^31^ In contrast to microstate topographies, these descriptors do not map the electrical activity in ‘the real (physical) 3D space’ but in an ‘abstract (mathematical) multidimensional space’, where each electrode represents one dimension.^31^ EEG dynamics are then understood as changes of state space vectors in this multidimensional space.^31^ Based on the dynamics of the state space vectors, a number of whole-brain electrical activity measures (the EEG state space descriptors, introduced by Wackermann in 1996^32^) exist: sigma is the total variance of the multichannel EEG over time.^33,34^ Phi is the normalized first derivative of the state space vector over time and yields an index of ‘central frequency’^33^ or ‘generalized frequency’^34^ of the EEG. Omega is the covariance structure of the set of underlying field generators and gives a lower-bound estimate of the number of uncorrelated processes in the analysed EEG,^33,34^ ranging from 1 to *k*, where *k* is the number of electrodes.^32^

Assessing the pattern of whole-brain electrical activity through appropriate analyses of multichannel EEG may thus be informative about the global organization and dynamics of neurocognitive networks throughout the entire course of anaesthesia.

It is not known whether microstate topographies in anaesthesia resemble microstate topographies in healthy resting states. It is further unknown whether microstate quantifiers and state space descriptors change with increasing anaesthesia duration. Even if these changes in whole-brain electrical activity EEG measures did occur, it is still unclear if these changes were different in patients with or without subsequent development of POD.

Therefore, the aim of this study was to compare microstate topographies under anaesthesia with those of healthy awake persons and to study microstate quantifiers as well as EEG global state space descriptors during anaesthesia in patients with and without subsequent POD. We assumed a pre-anaesthesia state of the brain such that the course of the whole-brain state of electrical activity during surgery would differ between those patients who did or did not subsequently develop POD—even if its onset occurred up to 7 days after the surgery. We were particularly interested in the changes with increasing anaesthesia duration in four groups varying in EEG suppression status and in subsequent POD status:

- Non-suppression EEG periods in patients without subsequent POD (Supp^−^|POD^−^, ‘intended course’).
- Suppression EEG periods in patients without subsequent POD (Supp^+^|POD^−^).
- Non-suppression EEG periods in patients with subsequent POD (Supp^−^|POD^+^).
- Suppression EEG periods in patients with subsequent POD (Supp^+^|POD^+^).

## Materials and methods

### Study population

The study population is a subsample of the randomized controlled Surgery Depth of Anaesthesia and Cognitive Outcome (SuDoCO) study, which evaluated the benefit of a bi-spectral index (BIS)–guided anaesthesia on the incidence of POD and postoperative cognitive decline. The SuDoCO study was originally approved in 2008 by the medical ethics committee of the Charité-Universitätsmedizin Berlin (EA1/242/08), registered in June 2009 with the ISRCTN registry (36437985), conducted between March 2009 and August 2010 and published in March 2013.^35^ Adult patients ≥ 60 years with planned elective surgery ≥ 60 min were included. Patients with cognitive impairment [Mini-Mental State Examination, (MMSE) < 24 points], a history of neurologic disease and patients engaged in a pharmaceutical study or patients not scheduled for general anaesthesia were excluded. The primary study outcome, POD, was evaluated twice daily through the seventh postoperative day. POD was assessed using the Nursing Delirium Screening Scale (NuDESC^36,37^) by trained medical personnel, unaware of the treatment group assignment. In a subsample of the SuDoCO study, the NuDESC showed a sensitivity of 0.98 and a specificity of 0.92 compared to the gold standard criteria of the Diagnostic and Statistical Manual of Mental Disorders (DSM-IV).^38^ Therefore, patients with a single or multiple positive delirium assessments were allocated to the ‘subsequent POD’ subgroup, while patients without a single positive delirium assessment were allocated to the ‘no subsequent POD’ subgroup.

During inclusion in the SuDoCO study, all patients were additionally asked to allow a multichannel EEG recording. Seventy-three consented. The multichannel intraoperative EEGs were not analysed so far, as they were not related to the primary research questions. The secondary data analyses presented here were approved by the medical ethics committee in February 2019 as an amendment to the original ethical committee approval in 2008.

### EEG measures

The intraoperative EEG was recorded throughout the entire surgical process. Acquisition EEG recordings were obtained for 19 electrode positions of the International 10/20 system, referenced to linked earlobes. Gold-plated cup electrodes were fixed, using paste and collodion, and the electrode impedances were kept below 5 kΩ. Additionally, an eye channel (electrooculogram) and an ECG channel (electrocardiogram) were used in order to detect artefacts. The TruScan32, produced by Deymed Diagnostic (Hronov, Czech Republic), with an amplifier bandpass of 0.16–450 Hz (1 dB points) with 50 Hz notch filtering was used as data acquisition system. The A/C converter sampled 4096 Hz per channel with a 14-bit resolution.

#### EEG data selection and preprocessing

The entire EEG traces were visually inspected. We intended to mark from shortly after induction until the end of the anaesthesia and every 30 min sets of 24 artefact-free EEG epochs of 2.5 s length, ideally grouped together as a one artefact-free 1-min EEG interval. In the case of suppression EEG or in the case of artefacts at the 30-min time slots, the nearest artefact-free non-suppression epochs were used. Assuming that the electric silence during intermittent low-amplitude EEG in burst suppression is harmful to the brain, only the (near-to-flat/low-amplitude) suppression epochs—but not the accompanying bursts—were selected within the entire record whenever they occurred. Suppression EEG was defined as periods of lower voltage with an amplitude of <10 µV.^39^ Twenty-four of these low-amplitude suppression epochs were summarized in one ‘suppression’ EEG interval. It turned out that suppression epochs were often shorter than 2.5 s, and thus, 24 epochs did not sum up to 1 min. In some cases, <24 artefact-free epochs were available. Therefore, the total time of each interval varied slightly and was shorter for suppression intervals than for non-suppression intervals.

The mean time since the induction of anaesthesia for each interval was defined as the average time since the induction of anaesthesia of all the 24 EEG epochs. This mean time elapsed from anaesthesia induction (‘anaesthesia duration’) was retained for the subsequent statistical modelling. ‘Anaesthesia duration’ for the suppression intervals was computed as described above for the non-suppression intervals. For the calculation of microstate topographies and microstate quantifiers, all selected EEG data were bandpass filtered from 2 to 20 Hz and recomputed to average reference. The overall distribution of all 885 analysed EEG intervals for all 73 patients is shown in Supplementary Fig. 1.

### Statistical analysis

#### Descriptive statistics of the patient sample

Nominal and ordinal data are presented as absolute and relative frequencies, while metric values, when normally distributed, are displayed as mean (standard deviation). Non-normally distributed variables are displayed as median (minimum–maximum). Differences between two independent groups were evaluated using *χ*^2^ test in analyses involving binary and ordinal variables. Student’s *t*-test and Mann–Whitney U-test were used in analyses involving continuous, normally distributed and continuous, non-normally distributed variables, respectively.

#### Quantitative EEG analysis

For the assessment of EEG microstates, in each set of selected 2.5 s epochs, time points of momentary maxima of the GFP were selected. In each of these sets, all selected maps were then submitted to a modified *k*-means clustering^40^ that ignored the EEG maps’ polarity and searched for four microstate class maps. This number of microstate classes was chosen in agreement with the majority of existing EEG microstate studies that had a similarly low number of electrodes, and in view of the need to limit the complexity of the expected result space, given the multifactorial nature of the data.^16^ All microstate computations were conducted using the EEG microstate plugin for EEGLAB developed by the last author and available here: https://www.thomaskoenig.ch/index.php/work/software/10-eeglab-plugin-manual.

In a next step, we computed group mean microstate maps for each patient group (with and without subsequent POD) and each selected interval, excluding time intervals that contained less than two data sets using an algorithm that again ignored map polarity, and that ordered the sequence of the microstate maps to be averaged such that the common topographic variance within microstate class was maximized.^41^ In the final step of the microstate cluster identification process, we applied the same algorithm to compute grand mean microstate class maps based on the time-conditional group mean maps. Finally, these grand mean microstate maps were labelled according to their similarity to template microstate class maps known from the literature.^22^ In order to test the obtained microstate maps for systematic differences depending on group and presence or absence of suppression, the corresponding individual microstate maps were submitted to a topographical ANOVA (TANOVA), with microstate class and presence/absence of suppression as within-subject factors and subsequent POD as between-subject factor. The TANOVA was limited to the first EEG intervals of each subject, as the currently available implementation of the TANOVA algorithm only permits for two within subject factors, such that time was omitted.^42^

To extract the microstate quantifiers, the maps of the individual EEG epochs that were at momentary peaks of the GFP were labelled based on the best-fitting grand mean microstate class. The assignment of the time periods in between GFP peaks was obtained by a nearest neighbour interpolation. EEG microstates were then defined as continuous time periods assigned to the same class. Microstates that were potentially truncated because they were observed at the beginning or the end of an EEG epoch were discarded. Finally, for each set of 2.5 s epochs, the mean duration, the mean frequency of occurrence and the mean GFP were computed. Microstate duration is ‘the average duration that a given microstate remains stable’,^16^ while frequency of occurrence is the ‘frequency of appearance of a given microstate within one time unit’. GFP is computed as the ‘mean of all absolute potential differences in the field corresponding to the spatial standard deviation’.^43^ Its value is independent of a reference location and quantifies the overall ‘synchronous activity of large numbers of intracranial neuronal elements’.^30^

The computation of the global state space descriptors in a 2.5-s EEG epoch was based on a spatial principal component analysis of the EEG data, which yielded a set of 19 eigenvectors and 19 eigenvalues, corresponding to the number of electrodes used in this study. After projecting the EEG onto these eigenvectors, a time trajectory of the EEG signal was produced through the state space spanned by these eigenvectors. Sigma was then computed as the sum of all eigenvalues, corresponding to the overall variance of the EEG data across all sampling electrodes and all time points within one interval.^34^ Phi was computed as the sum of the first derivative of the state space trajectory in time, normalized by sigma, which yields an index of generalized frequency of the EEG.^33,34^ Omega was computed as the Shannon entropy of the spectrum of the obtained eigenvalues, yielding a lower-bound estimate of the number of uncorrelated brain processes observed.^32,34^ Sigma, phi and omega values were averaged with each set of 24 EEG epochs per subject.

#### Multivariable analyses of the EEG parameters

The three microstate quantifiers duration, frequency of occurrence and GFP as well as the global EEG state space descriptors sigma, phi, and omega served as dependent variables in multivariable linear mixed-effects models. The main independent variables of interest were grouped in a three-way interaction between the mean time elapsed from anaesthesia induction (Variable 1: ‘anaesthesia duration’), whether or not the patients developed a subsequent POD (Variable 2: ‘subsequent POD’) and the current EEG status: non-suppression EEG interval versus suppression EEG interval (Variable 3: ‘suppression’). For the analysis of the microstate quantifiers, this three-way interaction term was expanded to a four-way interaction term by additionally including the variable microstate classes A–D (Variable 4: ‘microstates’). All models were adjusted for age, sex, cognitive status before surgery (MMSE 24–27/28–29/30 points), American Society of Anesthesiologists (ASA) Physical Status (ASA-PS I and II versus ASA-PS III and IV), site of surgery (thoracic and abdominal versus all other), type of anaesthetic [total intravenous anaesthesia (TIVA) versus inhalational anaesthesia] and randomization status. The model parameters were estimated using the ‘lmer’ function in the lmerTest package in R,^44^ and their significance was tested using R’s ‘aov’ function. The predicted values of the four EEG microstate quantifiers as well as the EEG state space descriptors were plotted as functions of anaesthesia duration, suppression and subsequent POD status using the ‘plot_model’ function in the sjPlot package in R.^45^

To answer the specific questions raised in the introduction, we set the reference of the linear mixed-effects models to no subsequent POD, no suppression and duration 0 and tested specific parameters of the obtained models (see Table 1).

**Table 1:** Parameter of interest in the models on EEG microstate quantifiers and on EEG state space descriptors

Model parameter in models with microstate quantifiers as dependent variables	Model parameter in models with EEG state space descriptors as dependent variables	Interpretation
anaesthesia duration + anaesthesia duration × microstates	anaesthesia duration
		Effect of anaesthesia duration in non-suppression EEG periods in patients without subsequent POD (Supp^−^|POD^−^, intended course)
anaesthesia duration +		Effect of anaesthesia duration in suppression EEG periods in patients without subsequent POD (Supp^+^|POD^−^)
anaesthesia duration × microstates +		
anaesthesia duration × suppression +	anaesthia duration + anaesthesia duration × suppression	
anaesthesia duration × microstates × suppression		
anaesthesia duration +		Effect of anaesthesia duration in non-suppression EEG periods in patients with subsequent POD (Supp^−^|POD^+^)
anaesthesia duration × microstates +		
anaesthesia duration × subsequent POD +	anaesthesia duration + anaesthesia duration × subsequent POD	
anaesthesia duration × microstates × subsequent POD		
anaesthesia duration + anaesthesia duration × microstates +		Effect of anaesthesia duration in suppression EEG periods in patients with subsequent POD (Supp^+^|POD^+^)
anaesthesia duration × suppression +	anaesthesia duration +	
anaesthesia duration × subsequent POD +	anaesthesia duration × suppression +	
anaesthesia duration × microstates × suppression +	anaesthesia duration × subsequent POD +	
anaesthesia duration × microstates × subsequent POD +	anaesthesia duration × suppression × subsequent POD	
anaesthesia duration × subsequent POD × suppression +		
anaesthesia duration × microstates × suppression × subsequent POD		

POD^−^, no postoperative delirium; POD^+^, postoperative delirium; Supp^−^, non-suppression EEG interval; Supp^+^, suppression EEG interval.

## Results

### Basic patients’ characteristics

The 73 older study participants (the patients’ characteristics are described in detail in the second column of Table 2) underwent a median anaesthesia duration of 3.5 h (range: 1.25–11 h). Overall, 720 EEG min were analysed, of which 210 min (29.2%) were in suppression. The 720 EEG min were derived from 885 EEG intervals. During seven postoperative days, 21 patients (29%) developed POD. When comparing study participants with and without subsequent POD (columns 3–5 in Table 2), those with subsequent POD were older and were more likely to have experienced abdominal and thoracic surgery. There was no difference in the number of analysed EEG intervals between patients with and without subsequent POD (*P* = 0.11).

**Table 2:** Basic patient characteristics and anaesthesia-related variables, all patients with multichannel EEG during anaesthesia and stratified by POD status, *n* = 73

Parameter	All patients	Patients without subsequent POD	Patients with subsequent POD	*P*
	(*n* = 73, 100%)	(*n* = 52, 71.2%)	(*n* = 21, 28.8%)	
Age in years, mean (SD)	70.7 (6.5)	69.6 (6.0)	73.4 (6.9)	0.022
Female sex, *n* (%)	29 (39.7)	21 (40.4)	8 (38.1)	0.86
MMSE in points, *n* = 72, median (range)	29 (24–30)	29 (24–30)	29 (24–30)	0.58
ASA physical status				
I and II	35 (47.9)	27 (51.9)	8 (38.1)	0.28
III and IV	38 (52.1)	25 (48.1)	13 (61.9)	
Surgical site				
Abdomen and thorax	25 (34.2%)	14 (26.9)	11 (52.4)	0.038
All other	48 (65.8%)	38 (73.1)	10 (47.6)	
Type of anaesthetic				
TIVA	26 (35.6%)	20 (38.5)	6 (28.6)	0.42
Inhalation	47 (64.4%)	32 (61.5)	15 (71.4)	
Duration of surgery in minutes, median (range)	130 (35–565)	125 (35–375)	145 (70–365)	0.18
Duration of anaesthesia in minutes, median (range)	213 (75–675)	200 (75–430)	240 (115–675)	0.13
Total number of EEG intervals	885	607	278	0.082^a^
Number of EEG intervals in non-suppression	591	413	178	
Number of EEG intervals in suppression	294	194	100	0.044^a^
Number of EEG intervals per patient, median (range)	10 (5–43)	10 (5–43)	11 (6–27)	0.11

POD, postoperative delirium; SD, standard deviation; MMSE, Mini-Mental State Examination; ASA, American Society of Anesthesiologists; TIVA, total intravenous anaesthesia.

^a^ *χ* ^2^ goodness-of-fit test.

### Microstate topographies

Overall, microstate topographies under anaesthesia (Fig. 1, second row) resembled those of healthy and much younger volunteers (Fig. 1, first row). The Pearson correlation coefficient across electrodes of all four microstates between the two groups varied only slightly between the four microstates. The lowest value was *r* = 0.845 for microstate class A. Of note, the normative topographies were derived from a sample of 496 healthy volunteers (230 females and 266 males) aged 6–80 years in an awake resting state. The volunteers were recruited from three distinct study centres (the USA, Cuba and Switzerland).^22^ When stratifying the four microstate topographies under anaesthesia across all non-suppression and suppression intervals (Fig. 1, third and fourth rows), microstate D topography showed the lowest correlation between non-suppression and suppression intervals (*r* = 0.71) and, for the suppression periods, the lowest correlation with normative microstate D topography (*r* = 0.76), whereas in non-suppression periods, microstate D topography was similar to the normative microstate D topography (*r* = 0.93).

**Figure 2:**
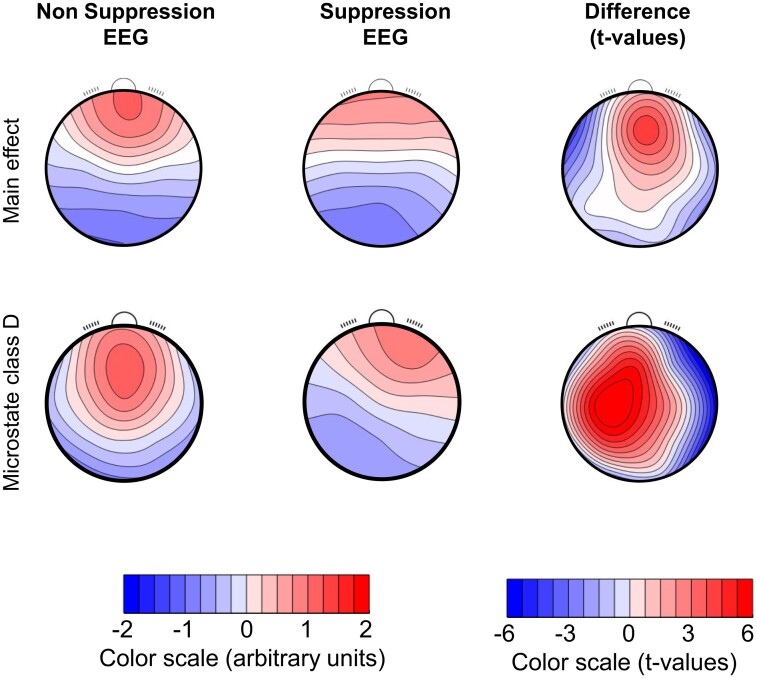
**Channel-wise comparison of the grand mean microstate topographies obtained in the first EEG interval without suppression against the first interval with suppression (*n* = 73 patients).** The upper row shows the main effect, i.e. the comparison of the average across all four microstate classes, and the lower row shows the comparison in microstate D. The left column shows the mean topographies for non-suppression EEG, the *middle* column shows the mean topographies for the data with suppression EEG and the right column shows t-maps comparing the two conditions. In the t-maps, the step between adjacent contour lines is 1t. Microstate D has been associated with the attention-related neural network in previous studies.

Conducting TANOVA on the individual microstate topographies (Fig. 2) yielded (as expected) a main effect of microstate class (*P* ≤ 0.0002), but also a main effect of presence of suppression (*P* ≤ 0.0002), and an interaction between the presence of suppression and the microstate class (*P* ≤ 0.0002). There were no significant main effects of subsequent POD or interactions that included subsequent POD. The main effect of ‘suppression’, obtained by comparing the non-suppression microstate topographies averaged across all classes with the average across the suppression-associated microstate topographies, was characterized by larger potentials in the non-suppression topographies at the midline frontal region (Fig. 2, upper row, right column). As also suggested by the spatial correlations shown in Fig. 1, the interaction between microstate class and presence of suppression was mostly driven by microstate class D (*r* = 0.710 between suppression and non-suppression intervals) that started to resemble microstate class C during suppression periods [indeed, among the four topographies obtained during non-suppression intervals, class C had the highest spatial correlation (*r* = 0.85) with the class D map obtained during suppression, data not shown]. The difference of the two template topographies was most pronounced at the right centro-parietal region (Fig. 2, bottom row, right column).

### Microstate quantifiers

All three investigated microstate quantifiers showed different trajectories over ongoing anaesthesia time, depending on whether patients with or without subsequent POD were exposed to anaesthesia and whether or not this happened during suppression EEG. This was reflected statistically by significant four-way interactions involving anaesthesia duration, subsequent POD status, EEG suppression status and microstates in models with microstate duration as well as frequency of occurrence as dependent variables and by a highly significant three-way interaction involving anaesthesia duration, subsequent POD status and EEG suppression status in models with microstate GFP as dependent variable (see Supplementary Table 1 with selected results of the ANOVA table for details).

**Figure 3:**
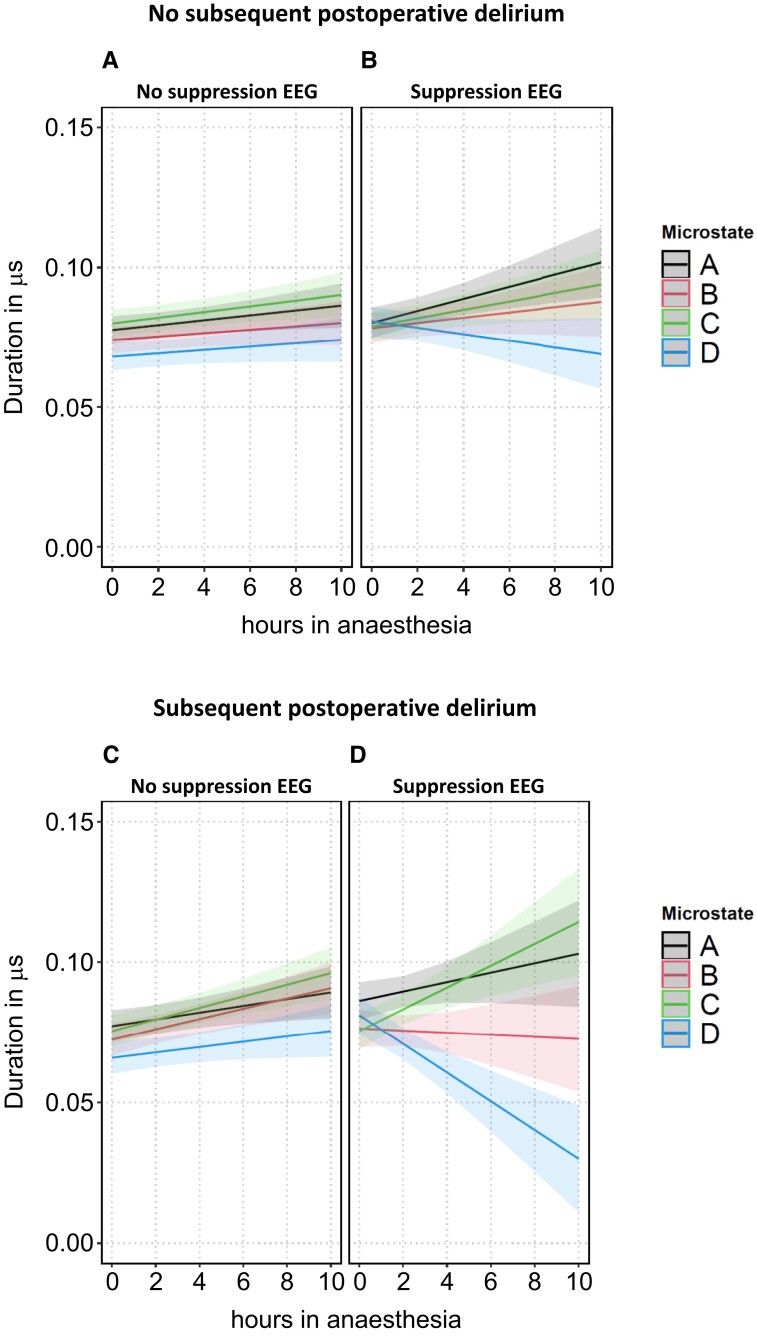
**Predicted values of the EEG microstate quantifier ‘duration’ in all microstates in patients without (panels A and B) and with (panels C and D) subsequent POD, stratified by suppression status, *n* = 73 patients.** The shaded bands around the predicted values represent the 95% prediction confidence intervals.

**Figure 4:**
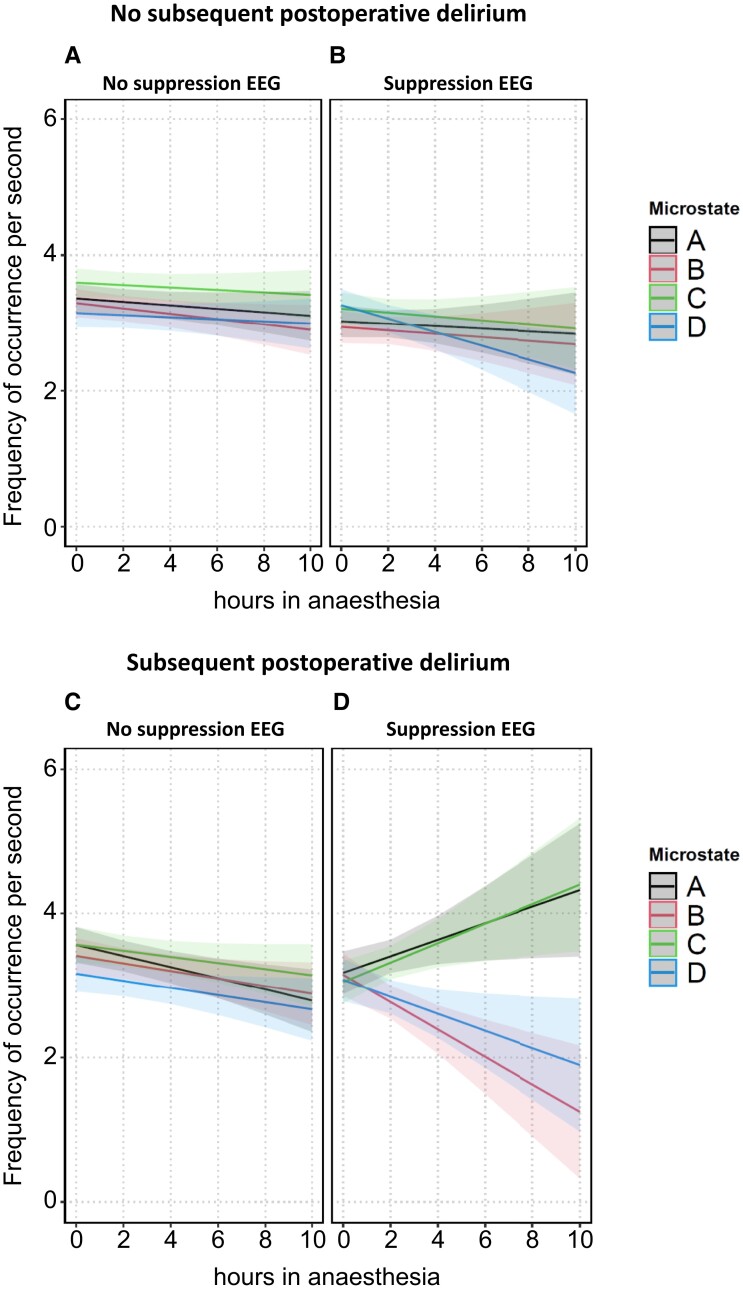
**Predicted values of the EEG microstate quantifier ‘frequency of occurrence’ in all microstates in patients without (panels A and B) and with (panels C and D) subsequent POD, stratified by suppression status, *n* = 73 patients.** The shaded bands around the predicted values represent the 95% prediction confidence intervals.

**Figure 5:**
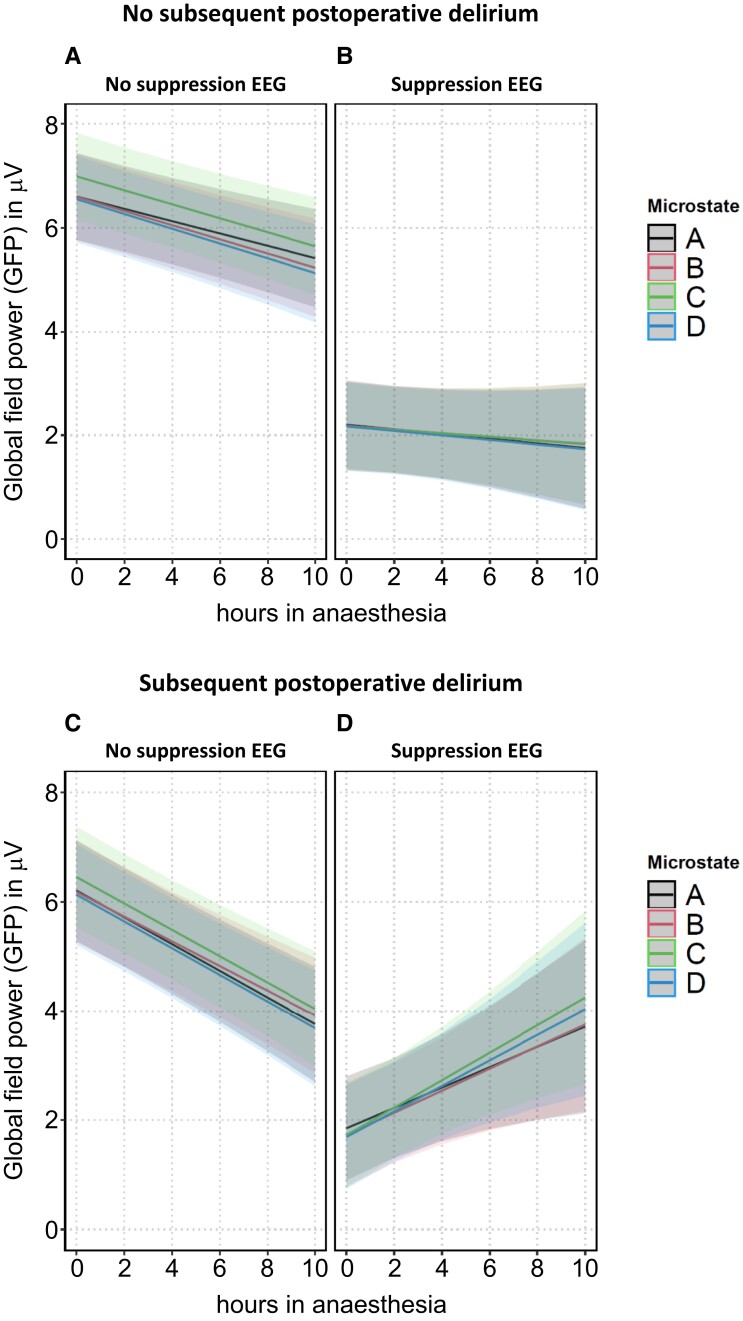
**Predicted values of the EEG microstate quantifier GFP in all microstates in patients without (panels A and B) and with (panels C and D) subsequent POD, stratified by suppression status, *n* = 73 patients.** The shaded bands around the predicted values represent the 95% prediction confidence intervals.

Under ongoing non-suppression EEG in patients without subsequent POD (Supp^−^|POD^−^, Figs 3–5, panel A), microstate duration increased (significantly in two out of four microstates), in parallel with a non-significant decrease in microstate frequency of occurrence. GFP decreased significantly in all four microstates. The changes of the three microstate quantifiers over ongoing anaesthesia time in non-suppression EEG remained synchronized across the four microstates. The corresponding coefficients of the three state space descriptors with their 95% confidence intervals and *P*-values are presented in Supplementary Tables 2–4 (‘anaesthesia duration + anaesthesia duration × microstates’).

Under ongoing suppression EEG in patients without subsequent POD (Supp^+^|POD^−^, Figs 3–5, panel B), microstate duration and frequency of occurrence showed a slight desynchronization with increasing time in anaesthesia: duration of microstates A and C significantly increased while duration of microstate D non-significantly decreased (*P* = 0.1). Frequency of occurrence of microstate D significantly decreased (*P* = 0.005) under suppression while there was no decrease under non-suppression EEG (*P* = 0.46). The offsets of GFP under suppression EEG were (as expected) significantly lower than when under non-suppression EEG. With continuing duration of anaesthesia, GFP showed a further, although non-significant, decline. The corresponding parameters of the three state space descriptors are presented in Supplementary Tables 2–4: ‘anaesthesia duration + anaesthesia duration × microstates + anaesthesia duration × suppression + anaesthesia duration × microstates × suppression’.

Continuing non-suppression EEG in patients with subsequent POD (Supp^−^|POD^+^, Figs 3–5, panel C) showed slight differences compared to the course of non-suppression EEG in patients without subsequent POD: the increase in microstate duration was more pronounced compared to patients with no subsequent POD with significant values in three out of four microstates and a non-significant increasing trend (*P* = 0.07) for microstate D. Frequency of occurrence decreased in all four microstates (significantly in microstates A and B, a non-significant decreasing trend in microstate D, *P* = 0.056, and non-significantly in microstate C), and the decline of GFP was more pronounced compared to patients with no subsequent POD (all *P* < 0.0001). The corresponding parameters are presented in Supplementary Tables 2–4: ‘anaesthesia duration + anaesthesia duration × microstates + anaesthesia duration × subsequent POD + anaesthesia duration × microstates × subsequent POD’.

For patients with subsequent POD and during suppression EEG (Supp^+^|POD^+^), the overall picture suggests strong desynchronization of microstates. The trajectories of microstate duration and frequency of occurrence no longer progressed synchronously within the four microstates: duration of microstate C increased (*P* = 0.0005) while duration of microstate D sharply decreased (*P* < 0.0001). Frequency of occurrence of microstate A (*P* = 0.0038) and of microstate C (*P* = 0.014) increased, while frequency of occurrence of microstate B (*P* = 0.0006) and of microstate D (*P* = 0.0033) decreased. GFP significantly increased in all four microstates with increasing anaesthesia duration, a course not detectable in any other combination of subsequent POD status and EEG status. The corresponding parameters are presented in the last rows of Supplementary Tables 2–4.

### EEG state space descriptors

As with the microstate quantifier, all three EEG state space descriptors showed different trajectories with increasing anaesthesia duration, depending on whether patients with or without subsequent POD were exposed and whether this exposure occurred during suppression or non-suppression EEG.

**Figure 6:**
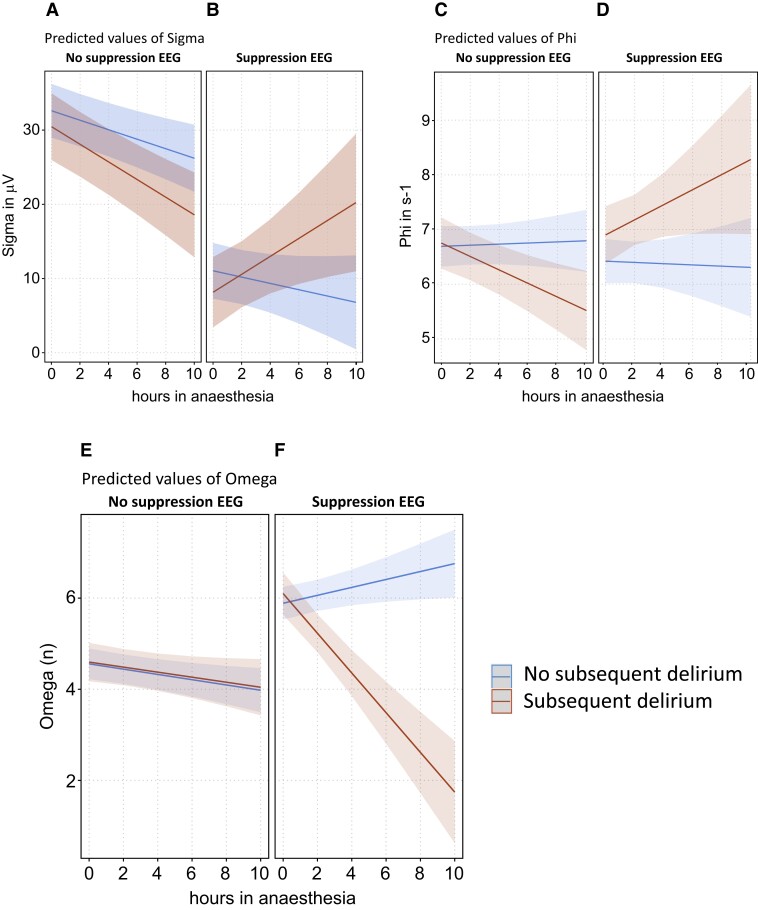
**Predicted values of the EEG state space descriptors in patients with and without subsequent POD, stratified by suppression status (panels A–F), *n* = 73 patients.** The shaded bands around the predicted values represent the 95% prediction confidence intervals.

Statistically, this was reflected by significant associations of all three EEG state space descriptors with the three-level interaction between anaesthesia duration, suppression status and subsequent POD status. Selected results of the ANOVA table derived from the multivariable linear mixed-effects models with the EEG state space descriptors as dependent variables are shown in Supplementary Table 5. The significant interactions are illustrated in Fig. 6, which shows the predicted values of the three EEG state space descriptors. The corresponding coefficients of anaesthesia duration in different patients’ groups are displayed in Supplementary Table 6.

With ongoing non-suppression EEG in patients without subsequent POD (Supp^−^|POD^−^), sigma and omega significantly decreased while phi showed no changes with increasing anaesthesia duration (blue lines in Fig. 6, panels A, C, and E). Under suppression (Supp^+^|POD^−^) and with increasing anaesthesia duration, sigma further non-significantly (*P* = 0.17) decreased (blue line in Fig. 6B), and omega strongly increased (*P* = 0.030, blue line in Fig. 6F). With ongoing non-suppression EEG periods in patients with subsequent POD (Supp^−^|POD^+^), sigma and phi significantly decreased (red lines in Fig. 6A and C) while omega showed a near-to-identical course as in patients without POD (red line in Fig. 6E). The combination of suppression EEG and subsequent POD (Supp^+^|POD^+^) led to a pronounced and significant increase in sigma (red line in Fig. 6B) and a pronounced and significant decrease in omega (red line in Fig. 6F). Phi, with ongoing suppression periods, increased by tendency (red line in Fig. 6D, *P* = 0.08).

## Discussion

### Statement of principal findings

Microstate topographies in older patients undergoing anaesthesia were overall comparable to those of healthy, awake and much younger persons except for microstate D topography under suppression. With increasing duration of exposure to anaesthesia, the trajectory of parameters of global electrical brain activity depended on whether non-suppression or suppression EEG occurred and whether or not a patient was vulnerable to subsequent POD. A picture of parallel evolution of the microstate quantifier duration and frequency of occurrence of epochs over ongoing anaesthesia time in non-suppression EEG changed in ongoing suppression EEG, particularly in patients with subsequent POD: duration of microstate D sharply decreased while duration of microstate C sharply increased. Likewise, frequency of occurrence of the four microstates evolved (partially non-significant) in opposite directions. Sigma, parallel with GFP, and—by tendency—the generalized frequency phi increased, a pattern not detectable in any other combination of EEG status and subsequent POD status. Additionally, the number of uncorrelated brain processes (omega) sharply decreased.

### Changes of microstate quantifier with increasing exposure to anaesthesia

Several studies identified suppression and especially cumulative suppression as a risk factor for POD.^46-55^ The analyses presented here identified a number of changes in whole-brain electrical activity, which occurred with increasing duration of anaesthesia in suppression EEG but not in non-suppression EEG. One putatively adverse reaction is the destabilized course of microstate duration and frequency of occurrence with increasing duration of exposure to anaesthesia. In ongoing non-suppression EEG, duration and frequency of occurrence in all four microstates changed in parallel. This parallelism was no longer present in suppression EEG. Microstate duration and frequency of occurrence, which were initially quite similar between classes, began to diverge markedly in patients with subsequent POD. More specifically, duration of microstate class D became significantly briefer and microstate D occurred less frequently over time.

POD, especially in its hyperactive form, shares agitation, psychomotor restlessness and ‘positive psychotic features’ such as hallucinations and illusions^56^ with disease conditions such as schizophrenia. EEG studies in (awake) patients with schizophrenia used a four-microstate topography solution comparable to the solution in the data presented here. Their cross-sectional comparisons with healthy subjects observed the patients with schizophrenia to have decreased duration^41,57-61^ and lower frequency of occurrence^59,62,63^ in microstate D. Of note, these studies also observed in patients with schizophrenia prolonged duration^59,62-64^ and higher frequency of occurrence^57-59,63^ in microstate C. Thus, changes in quantifiers in microstates D and C occurred within repeated suppression EEG intervals with increasing exposure to anaesthesia in patients with subsequent POD (Supp^+^|POD^+^) that resemble neural network features seen in awake patients experiencing schizophrenic episodes.

In addition, the analyses of the microstate class topographies indicated that during suppression EEG intervals, microstate class D topography shifted away from its typical configuration. This qualitative alteration of topography in microstate D did not occur in non-suppression conditions. Our data thus suggest that it is this class D microstate topography that is far more affected by exposure to excessively deep anaesthesia over time than the remaining microstate classes.

Microstate D has been linked to a number of underlying brain sources: Custo *et al*.,^28^ using a seven-microstate solution, found microstate D (its topography resembled microstate D in the four-microstate solution presented in Fig. 1, second row) associated with activities in the right inferior parietal lobe [Brodmann area (BA) 40], in the right middle and superior frontal gyri and in the right insula (BA 13). One study evaluated decreased activity in the right inferior parietal lobules and in the superior parietal lobules in post-stroke delirium.^65^ A source analysis with low-resolution electromagnetic tomography (LORETA) revealed the anterior cingulate cortex and additional right fronto-temporal brain areas (the latter again correlated with microstate D^16^) as being associated with post-electroconvulsive therapy delirium.^66^ In addition, Custo *et al*.^28^ linked microstate C to the precuneus and the posterior cingulate cortex (PCC). The PCC plays an important role in the default mode network: in the awake state, it is active during internally directed thought and inactive during external, task-directed attention.^67^ But in delirium, the dorsolateral prefrontal cortex (DLPFC) and PCC become strongly correlated, therefore indicating a disruption of the normal anti-correlation between the executive (DLPFC) and default mode networks (PCC).^68^ Consistent with this, van Montfort *et al*.^69^ found a less efficient resting state network (in delirious patients) and less connectivity in the right posterior cingulate while others have also observed disrupted network connectivity and network disintegration.^70,71^ These functional states seem likely to reflect acute disruption of networks that may already be vulnerable preoperatively as suggested by human diffusion tensor imaging studies^72^ and by both human and mouse studies reflecting disrupted synaptic structures associated with delirium in humans^73^ and vulnerability to delirium-like deficits in mice.^74^

### Changes of state space descriptors with increasing exposure to anaesthesia

Not only had the microstate quantifiers changed over time, the state space descriptors also indicated a pattern of potentially harmful changes over continuing suppression EEG intervals. These were associated with a drastically increased monotony in whole-brain electrical activity. While the brains of patients without subsequent POD kept the generalized frequency (phi) stable and even augmented the number of uncorrelated brain processes (omega) with ongoing suppression intervals, the brains of patients with subsequent POD experienced a sharp decline in omega. These changes accompanied an increase in overall EEG variance, reflected by a sharp and significant increase in both sigma and in the microstate quantifier GFP. Likewise, the generalized frequency increased, a pattern not detectable in any other of the three combinations of EEG status and subsequent POD status. A reasonable explanation for these results is that they reflect a loss in coping ability of the brain that leads to detrimental aftereffects that result in the onset of POD. Of note, a similar pattern of increasingly strong and increasingly synchronized neuronal discharge signal amplitude was observed when the brain transitioned from an interictal to an ictal state during epilepsy.^75^

### Strengths and weaknesses of this study in relation to other studies

Although several studies applied multichannel EEG recordings during anaesthesia,^76-81^ none analysed microstates or global state space descriptors over several hours of exposure to anaesthesia. One open-source EEG recording data set of the University of Cambridge came from an experimental study involving 20 healthy volunteers. The study applied high-density EEG recordings in four 7-min intervals during alertness, then light sedation to moderate sedation and finally back to recovery. These data were used to compare microstate solutions derived from different numbers of channels (from 8 to 91). The authors concluded that electrode densities > 20 are necessary to obtain reliable results because they discovered consistencies between microstate solutions derived from 32, 64 and 91 channels but not with the lower resolutions with 8 and 19 channels.^79^ Thus, the 19-channel solution used in our analysis is slightly below this threshold, which may limit the validity of our findings. The same data set was used to compare microstate topographies and quantifiers between baseline state (alertness) and moderate sedation state.^81^ The authors used a five-microstate solution with microstate topographies A–D comparable to our solution. The authors evaluated no differences in microstate duration between both states but found differences in frequency of occurrence in one microstate and a difference in coverage (the proportion of appearance of a certain microstate in a whole sequence) in another microstate.^81^ Another study evaluated microstates and their quantifiers during the first minutes after introduction of a propofol-induced anaesthesia in patients undergoing minor elective ear–nose–throat surgery.^80^ The authors used a five-microstate solution and found in four out of five microstate topographies a high correlation (>0.95) between the microstate topographies at baseline in the fully awake patients and the deeply sedated patients, who no longer responded to noxious stimuli.^80^ Our data confirmed these high correlations of overall microstate topographies following exposure to anaesthesia with those of healthy (awake) persons, but we found that these high correlations did not persist during suppression EEG intervals, especially in patients with subsequent POD.

### Limitations

Because of the real-world setting in an operating room (hammering, electrocautery to stop bleedings), the raw multichannel EEG showed multiple artefacts. Therefore, the overall duration and proportion of suppression and non-suppression EEG epochs in each patient remain indeterminate. In addition, only 19 channels of EEG were available, which constrained us to limit the microstate analysis to four classes. Furthermore, general anaesthesia in this study was a mixture of TIVA and inhalational anaesthesia. There is evidence that the EEG patterns vary between these two types of anaesthetics.^82,83^ As just 26 study participants received TIVA and inhalational anaesthesia was a mix of different regimens (isoflurane, desflurane or sevoflurane), a sensitivity analysis involving different types of anaesthesia was deemed infeasible.

POD develops up to several days after surgery.^6,7^ The EEG intervals in the analyses presented here encompassed neither EEG in the post-anaesthesia care unit nor in the hours or days before the onset of delirium symptoms. Thus, we do not know if the observed differences in microstates as well as in global EEG parameters during anaesthesia between patients with and without subsequent POD persist after surgery and contribute to the onset of POD. The relevant changes in global brain activity, which are causal for POD, may conceivably develop well after the surgery/anaesthesia has ended.

Future studies with higher-density EEG, larger patient groups and EEG recordings beyond the operating room may overcome these limitations.

The multichannel EEG and the parallel mounted BIS monitor were two technically different devices with no master clock. This lack of a master clock prevented the authors from obtaining the actual BIS values for each of the (multichannel) EEG epochs that would have allowed them to adjust the multivariable linear mixed-effects models for depth of anaesthesia. Therefore, the multivariable model was adjusted only for the randomization status (BIS open/BIS blinded) as a crude proxy for the overall course of the depth of anaesthesia in each patient. This limitation may be resolved through technical solutions in future studies.

### Meaning of the study

Our analyses indicate that even under non-suppression EEG, continuing anaesthesia duration has effects on whole-brain electrical activity. A resilient brain seems to be able to maintain a stable organization with only slight changes in the duration and frequency of occurrence of the microstates. Likewise, the generalized frequency and the spatial complexity seem to be resilient even after hours of exposure to anaesthesia. However, in patients with subsequent POD, this resilience appears to be already weakened during ongoing non-suppression EEG periods and is particularly and increasingly disrupted during periods of EEG suppression. Hence, under these conditions, the overall complexity of the brain declines dramatically and exhibits a combination of relatively few uncorrelated brain processes with a large variance and increased generalized frequency. Furthermore, brain network functions that were previously found to be protective against delusional states in schizophrenia appear to be especially impaired, which might contribute to the subsequent appearance of POD. As POD develops up to several days after the end of anaesthesia/surgery, the different trajectories in whole-brain electrical activity during anaesthesia may thus be seen as biomarkers of brain vulnerability to POD. While the brain of patients without subsequent POD may still be able to maintain its overall functioning outside the operating room and the perioperative setting, the combined stresses of anaesthetics, inflammation and physiologic disturbances during surgery may evoke the global brain aberrations in those patients who subsequently develop POD.

## Supplementary Material

fcad270_Supplementary_Data

## Data Availability

All data are available in the main text or the Supplementary materials. The EEGLAB microstate plugin for the analysis of the microstate topographies (Fig. 1) as well as the Ragu software to analyse the data as presented in Fig. 2 are available upon request from T.K. at https://www.thomaskoenig.ch/index.php/work/software/ragu. The R script to run the multivariable linear mixed-effects models is available upon request from B.N.
