# Supplementary material for: EEG microstate quantifiers and state space descriptors during anaesthesia in patients with postoperative delirium: a descriptive analysis
Source: Brain Commun. 2023 Oct 17;5(6):fcad270. doi: 10.1093/braincomms/fcad270 (PMC10629467; doi:10.1093/braincomms/fcad270)
Supplement: fcad270_Supplementary_Data [file fcad270_supplementary_data.docx]

Supplementary Materials for

**EEG microstate quantifiers and state space descriptors during anaesthesia in patients with postoperative delirium: a descriptive analysis**

Supplementary Fig. 1 Distribution of the selected 885 artefact-free EEG intervals, stratified by type of EEG interval, n = 73 patients


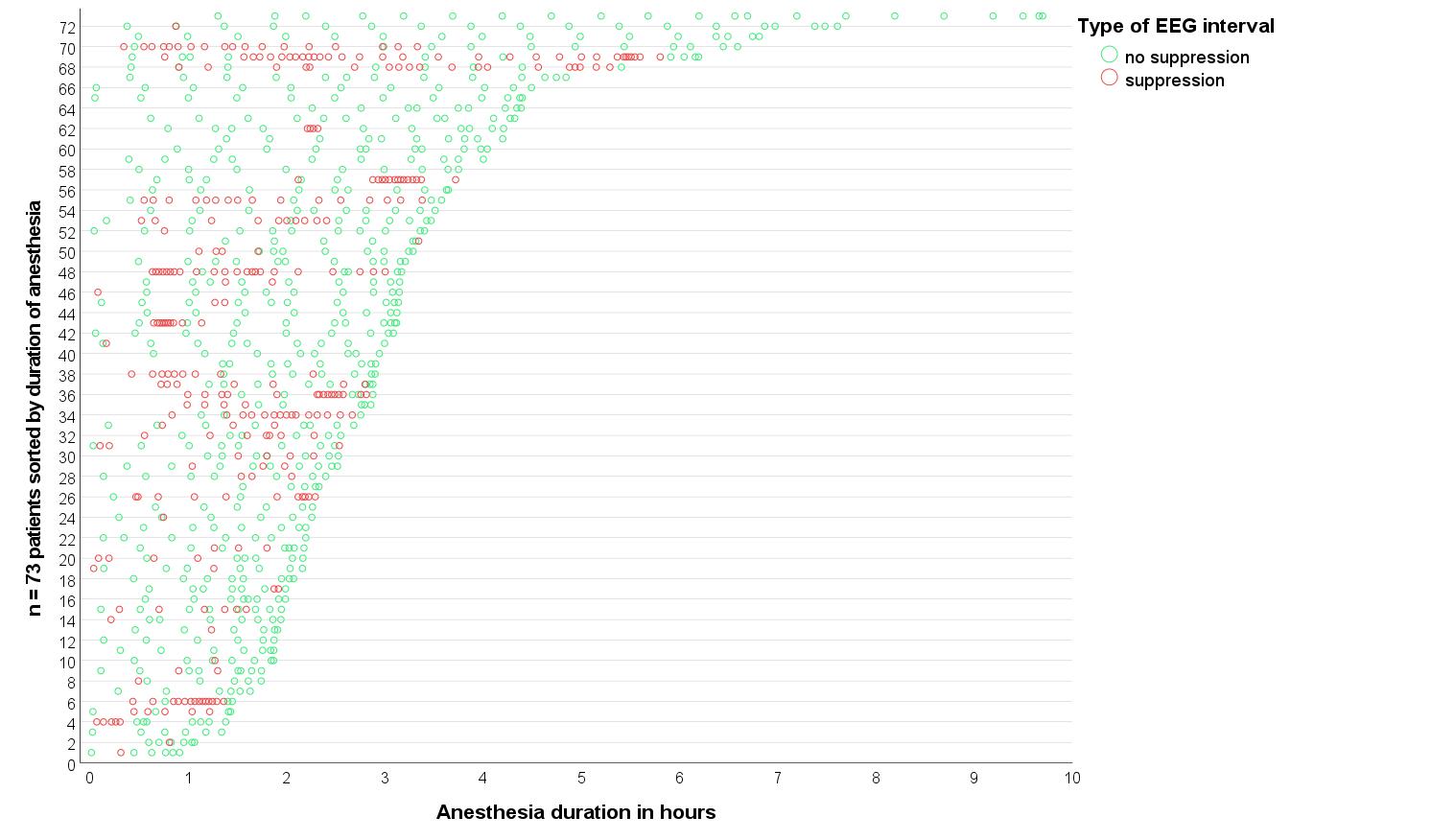


**Supplementary Table 1: ANOVA table of the EEG microstate quantifiers (duration, frequency of occurrence and global field power) in multichannel EEG after exposure to anaesthesia, n = 73**

| **Parameter** | **duration** | | **frequency of occurrence** | | **global field power** | |
| --- | --- | --- | --- | --- | --- | --- |
|  | ***F* value** | ***P*-value** | ***F* value** | ***P*-value** | ***F* value** | ***P*-value** |
| microstates | 11.48 | < 0.00001 | 4.59 | 0.003 | 2.18 | 0.089 |
| anaesthesia duration | 12.05 | 0.0005 | 9.44 | 0.002 | 8.58 | 0.003 |
| suppression | 37.11 | < 0.00001 | 34.68 | <0.00001 | 4326.22 | <0.00001 |
| subsequent POD | 0.18 | 0.67 | 0.19 | 0.67 | 1.22 | 0.27 |
| age | 0.37 | 0.54 | 0.55 | 0.46 | 6.68 | 0.012 |
| sex | 4.63 | 0.035 | 4.43 | 0.039 | 12.07 | 0.0009 |
| randomization status | 0.001 | 0.97 | 0.22 | 0.64 | 9.43 | 0.003 |
| MMSE | 1.91 | 0.16 | 2.17 | 0.12 | 0.08 | 0.92 |
| ASA PS | 5.25 | 0.025 | 4.86 | 0.031 | 0.06 | 0.81 |
| site of surgery | 0.08 | 0.78 | 0.004 | 0.95 | 0.07 | 0.80 |
| type of anaesthetic | 9.39 | 0.003 | 11.34 | 0.0013 | 15.52 | 0.00021 |
| anaesthesia duration*microstates | 1.08 | 0.36 | 7.18 | 0.00008 | 0.07 | 0.97 |
| anaesthesia duration*suppression | 2.72 | 0.099 | 0.29 | 0.59 | 80.02 | <0.00001 |
| anaesthesia duration*subsequent POD | 3.27 | 0.071 | 0.004 | 0.95 | 5.30 | 0.02 |
| suppression*microstates | 13.65 | <0.00001 | 6.42 | 0.0002 | 2.54 | 0.055 |
| subsequent POD*microstates | 2.98 | 0.03 | 3.46 | 0.016 | 0.17 | 0.92 |
| subsequent POD*suppression | 1.61 | 0.20 | 0.63 | 0.43 | 0.007 | 0.93 |
| anaesthesia duration*suppression*microstates | 11.35 | <0.00001 | 7.44 | 0.000058 | 0.18 | 0.91 |
| anaesthesia duration*subsequent POD*microstates | 4.29 | 0.005 | 4.31 | 0.005 | 0.12 | 0.95 |
| anaesthesia duration*subsequent POD*suppression | 3.33 | 0.068 | 1.97 | 0.16 | 34.76 | <0.00001 |
| subsequent POD*suppression*microstates | 0.80 | 0.49 | 0.62 | 0.60 | 0.09 | 0.96 |
| anaesthesia duration*subsequent POD*suppression*microstates | 3.03 | 0.028 | 5.63 | 0.00075 | 0.08 | 0.97 |

ANOVA table of the fully adjusted models with 4-way interaction between anaesthesia duration*microstates*suppression*subsequent POD, n = 73.

POD = postoperative delirium status; MMSE = Mini–Mental State Examination; ASA PS = American Society of Anesthesiologists physical status classification.

**Supplementary Table 2: Effect sizes and their 95% confidence intervals in anaesthesia duration in different patient groups. Results of multiple adjusted linear mixed models with the EEG microstate quantifier *duration* as dependent variable, n = 73**

| **Parameter (effect per additional hour anaesthesia duration)** | **Interpretation** | **Microstate** | **estimate (95% confidence interval), *P* -value** |
| --- | --- | --- | --- |
| anaesthesia duration +  anaesthesia duration × microstates | effect of anaesthesia duration in different microstates in non-suppression EEG periods in patients without subsequent postoperative delirium (Supp-\|POD-, intended course), | A | 0.00088 (0.00005 to 0.0017), *P =* 0.038 |
|  |  | B | 0.00061 (-0.00021 to 0.0014), *P =* 0.15 |
|  |  | C | 0.0010 (0.00020 to 0.0019), *P =* 0.015 |
|  |  | D | 0.00061 (-0.00022 to 0.0014), *P =* 0.15 |
| anaesthesia duration +  anaesthesia duration × microstates +  anaesthesia duration × suppression +  anaesthesia duration × microstates × suppression | effect of anaesthesia duration in suppression EEG periods in patients without subsequent postoperative delirium (Supp+\|POD-), | A | 0.00216 (0.00075 to 0.0036), *P =* 0.0027 |
|  |  | B | 0.00093 (-0.00048 to 0.0023), *P =* 0.29 |
|  |  | C | 0.0015 (0.00009 to 0.0029), *P =* 0.0037 |
|  |  | D | -0.00117 (-0.0026 to 0.00024), *P =* 0.10 |
| anaesthesia duration +  anaesthesia duration × microstates +  anaesthesia duration × subsequent POD +  anaesthesia duration × microstates × subsequent POD | effect of anaesthesia duration in non-suppression EEG periods in patients with subsequent postoperative delirium (Supp-\|POD+), and | A | 0.0012 (0.00017 to 0.0022), *P =* 0.022 |
|  |  | B | 0.0018 (0.00081 to 0.0029), *P =* 0.0005 |
|  |  | C | 0.0021 (0.0010 to 0.0031), *P =* 0.0001 |
|  |  | D | 0.00096 (-0.00008 to 0.0020), *P =* 0.069 |
| anaesthesia duration +  anaesthesia duration × microstates +  anaesthesia duration × suppression +  anaesthesia duration × subsequent POD +  anaesthesia duration × microstates × suppression +  anaesthesia duration × microstates × subsequent POD +  anaesthesia duration × subsequent POD × suppression +  anaesthesia duration × microstates × suppression × subsequent POD | effect of anaesthesia duration in suppression EEG periods in patients with subsequent postoperative delirium (Supp+\|POD+). | A | 0.00168 (-0.0052 to 0.0039), *P =* 0.13 |
|  |  | B | -0.00037 (-0.0026 to 0.0018), *P =* 0.74 |
|  |  | C | 0.0039 (0.0017 to 0.0061), *P =* 0.0005 |
|  |  | D | -0.0051 (-0.0073 to -0.0029), *P <* 0.0001 |

POD = postoperative delirium; Supp- = non-suppression EEG interval; Supp+ = suppression EEG interval; POD- = no postoperative delirium; POD+ = postoperative delirium.

**Supplementary Table 3: Effect sizes and their 95% confidence intervals in anaesthesia duration in different patient groups. Results of multiple adjusted linear mixed models with the EEG microstate quantifier *frequency of occurrence* as dependent variable, n = 73**

| **Parameter (effect per additional hour anaesthesia duration)** | **Interpretation** | **Microstate** | **estimate (95% confidence interval), *P* -value** |
| --- | --- | --- | --- |
| anaesthesia duration +  anaesthesia duration × microstates | effect of anaesthesia duration in different microstates in non-suppression EEG periods in patients without subsequent postoperative delirium (Supp-\|POD-, intended course), | A | -0.026 (-0.066 to 0.015), p=0.22 |
|  |  | B | -0.040 (-0.080 to 0.0011), p=0.056 |
|  |  | C | -0.018 (-0.059 to 0.023), p=0.39 |
|  |  | D | -0.015 (-0.056 to 0.025), p=0.46 |
| anaesthesia duration +  anaesthesia duration × microstates +  anaesthesia duration × suppression +  anaesthesia duration × microstates × suppression | effect of anaesthesia duration in suppression EEG periods in patients without subsequent postoperative delirium (Supp+\|POD-), | A | -0.018 (-0.087 to 0.051), p=0.61 |
|  |  | B | -0.025 (-0.094 to 0.045), p=0.48 |
|  |  | C | -0.029 (-0.098 to 0.041), p=0.42 |
|  |  | D | -0.10 (-0.169 to -0.031), p=0.0048 |
| anaesthesia duration +  anaesthesia duration × microstates +  anaesthesia duration × subsequent POD +  anaesthesia duration × microstates × subsequent POD | effect of anaesthesia duration in non-suppression EEG periods in patients with subsequent postoperative delirium (Supp-\|POD+), and | A | -0.078 (-0.129 to -0.027), p=0.0028 |
|  |  | B | -0.053 (-0.104 to -0.002), p=0.042 |
|  |  | C | -0.043 (-0.094 to 0.0081), p=0.10 |
|  |  | D | -0.050 (-0.10 to 0.001), p=0.056 |
| anaesthesia duration +  anaesthesia duration × microstates +  anaesthesia duration × suppression +  anaesthesia duration × subsequent POD +  anaesthesia duration × microstates × suppression +  anaesthesia duration × microstates × subsequent POD +  anaesthesia duration × subsequent POD × suppression +  anaesthesia duration × microstates × suppression × subsequent POD | effect of anaesthesia duration in suppression EEG periods in patients with subsequent postoperative delirium (Supp+\|POD+). | A | 0.115 (0.006 to 0.223), p=0.038 |
|  |  | B | -0.19 (-0.299 to -0.08), p=0.0006 |
|  |  | C | 0.135 (0.027 to 0.243), p=0.014 |
|  |  | D | -0. 118 (-0.226 to -0.0097), p=0.033 |

POD = postoperative delirium; Supp- = non-suppression EEG interval; Supp+ = suppression EEG interval; POD- = no postoperative delirium; POD+ = postoperative delirium.

**Supplementary Table 4: Effect sizes and their 95% confidence intervals in anaesthesia duration in different patient groups. Results of multiple adjusted linear mixed models with the EEG microstate quantifier *global field power* as dependent variable, n = 73**

| **Parameter (effect per additional hour anaesthesia duration)** | **Interpretation** | **Microstate** | **estimate (95% confidence interval), *P* -value** |
| --- | --- | --- | --- |
| anaesthesia duration +  anaesthesia duration × microstates | effect of anaesthesia duration in different microstates in non-suppression EEG periods in patients without subsequent postoperative delirium (Supp-\|POD-, intended course), | A | -0.119 (-0.177 to -0.060), p=0.0001 |
|  |  | B | -0.136 (-0.195 to -0.078), p<0.0001 |
|  |  | C | -0.134 (-0.193 to -0.076), p<0.0001 |
|  |  | D | -0.142 (-0.201 to -0.083), p<0.0001 |
| anaesthesia duration +  anaesthesia duration × microstates +  anaesthesia duration × suppression +  anaesthesia duration × microstates × suppression | effect of anaesthesia duration in suppression EEG periods in patients without subsequent postoperative delirium (Supp+\|POD-), | A | -0.045 (-0.145 to 0.056), p=0.38 |
|  |  | B | -0.035 (-0.135 to 0.066), p=0.50 |
|  |  | C | -0.032 (-0.133 to 0.068), p=0.53 |
|  |  | D | -0.044 (-0.144 to 0.057), p=0.39 |
| anaesthesia duration +  anaesthesia duration × microstates +  anaesthesia duration × subsequent POD +  anaesthesia duration × microstates × subsequent POD | effect of anaesthesia duration in non-suppression EEG periods in patients with subsequent postoperative delirium (Supp-\|POD+), and | A | -0.245 (-0.319 to -0.171), p<0.0001 |
|  |  | B | -0.226 (-0.300 to -0.152), p<0.0001 |
|  |  | C | -0.241 (-0.315 to -0.167), p<0.0001 |
|  |  | D | -0.245 (-0.318 to -0.171), p<0.0001 |
| anaesthesia duration +  anaesthesia duration × microstates +  anaesthesia duration × suppression +  anaesthesia duration × subsequent POD +  anaesthesia duration × microstates × suppression +  anaesthesia duration × microstates × subsequent POD +  anaesthesia duration × subsequent POD × suppression +  anaesthesia duration × microstates × suppression × subsequent POD | effect of anaesthesia duration in suppression EEG periods in patients with subsequent postoperative delirium (Supp+\|POD+). | A | 0.186 (0.030 to 0.343), p=0.020 |
|  |  | B | 0.202 (0.046 to 0.359), p=0.011 |
|  |  | C | 0.252 (0.095 to 0.409), p=0.002 |
|  |  | D | 0.234 (0.077 to 0.391), p=0.0035 |

POD = postoperative delirium; Supp- = non-suppression EEG interval; Supp+ = suppression EEG interval; POD- = no postoperative delirium; POD+ = postoperative delirium.

**Supplementary Table 5: ANOVA table of the EEG state space descriptors (Sigma, Phi and Omega) in multichannel EEG during anaesthesia, n = 73**

| **Parameter** | **Sigma** | | **Phi** | | **Omega** | |
| --- | --- | --- | --- | --- | --- | --- |
|  | ***F* value** | ***P*-value** | ***F* value** | ***P*-value** | ***F* value** | ***P*-value** |
| anaesthesia duration | 2.35 | 0.12 | 0.01 | 0.90 | 27.96 | <0.00001 |
| suppression | 946.31 | <0.00001 | 0.31 | 0.58 | 239.13 | <0.00001 |
| subsequent POD | 1.44 | 0.23 | 1.49 | 0.22 | 0.40 | 0.53 |
| Age | 6.27 | 0.015 | 0.001 | 0.97 | 2.55 | 0.11 |
| Sex | 11.91 | 0.001 | 1.62 | 0.21 | 0.24 | 0.63 |
| randomization status | 8.03 | 0.0062 | 0.0007 | 0.98 | 0.37 | 0.54 |
| MMSE | 0.10 | 0.90 | 1.42 | 0.25 | 0.92 | 0.40 |
| ASA PS | 0.13 | 0.72 | 7.06 | 0.010 | 0.07 | 0.79 |
| Site of surgery | 0.04 | 0.84 | 0.17 | 0.68 | 0.09 | 0.77 |
| type of anaesthetic | 13.89 | 0.0004 | 6.20 | 0.015 | 2.52 | 0.12 |
| anaesthesia duration*suppression | 16.53 | 0.00005 | 5.53 | 0.019 | 8.15 | 0.0044 |
| anaesthesia duration*subsequent POD | 2.53 | 0.11 | 0.021 | 0.88 | 35.48 | <0.00001 |
| suppression* subsequent POD | 0.28 | 0.60 | 3.41 | 0.065 | 0.89 | 0.35 |
| anaesthesia duration*suppression*subsequent POD | 11.56 | 0.0007 | 7.72 | 0.006 | 40.68 | <0.00001 |

ANOVA table of the fully adjusted models with 3-way interaction between anaesthesia duration*suppression*subsequent POD, n = 73.

POD = postoperative delirium status; MMSE = Mini–Mental State Examination; ASA PS = American Society of Anesthesiologists physical status classification.

**Supplementary Table 6: Effect sizes and their 95% confidence intervals of anaesthesia duration in the EEG state space descriptors Sigma, Phi and Omega in different patient groups, n = 73**

| **Parameter** | **Interpretation** | **Sigma** | **Phi** | **Omega** |
| --- | --- | --- | --- | --- |
|  |  | ***estimate*** ‘***per additional hour in anaesthesia’ (95% confidence interval), P*-*value*** | | |
| anaesthesia duration | effect of anaesthesia duration in non-suppression EEG periods in patients without subsequent postoperative delirium (Supp-\|POD-, intended course) | -0.64 (-1.00 to -0.28),  *P* = 0.0005 | - 1. (-0.05 to 0.07),   2. *P* = 0.73 | -0.06 (-0.10 to -0.01),  *P* = 0.013 |
| anaesthesia duration +  anaesthesia duration*suppression | effect of anaesthesia duration in suppression EEG periods in patients without subsequent postoperative delirium (Supp+\|POD-) | -0.43 (-1.04 to 0.19),  *P* = 0.17 | -0.01 (-0.11 to 0.09),  *P* = 0.82 | 0.09 (0.01 to 0.17),  *P* = 0.030 |
| anaesthesia duration +  anaesthesia duration*subsequent POD | effect of anaesthesia duration in non-suppression EEG periods in patients with subsequent postoperative delirium (Supp-\|POD+) | -1.19 (-1.67 to -0.71),  *P* < 0.0001 | -0.12 (-0.20 to -0.05),  *P* = 0.0015 | -0.06 (-0.12 to 0.01),  *P* = 0.080 |
| anaesthesia duration +  anaesthesia duration*suppression +  anaesthesia duration* subsequent POD +  anaesthesia duration*suppression* subsequent POD | effect of anaesthesia duration in suppression EEG periods in patients with subsequent postoperative delirium (Supp+\|POD+) | 1.21 (0.23 to 2.19),  *P* = 0.016 | 0.14 (-0.02 to 0.29),  *P* = 0.081 | -0.44 (-0.56 to -0.31),  *P* < 0.0001 |

Results of multiple adjusted linear mixed models with the EEG state space descriptors Sigma, Phi and Omega as dependent variables, n = 73. POD = postoperative delirium status; Supp- = non-suppression EEG interval; Supp+ = suppression EEG interval; POD- = no postoperative delirium; POD+ = postoperative delirium.
